# Reliability generalization meta-analysis of Cronbach’s alpha of the oral impacts on daily performance (OIDP) questionnaire

**DOI:** 10.1186/s12903-025-05496-3

**Published:** 2025-02-11

**Authors:** Kalyana Chakravarthy Pentapati, Deepika Chenna, Vijay S. Kumar, Nanditha Kumar

**Affiliations:** 1 Department of Public Health Dentistry, Manipal College of Dental Sciences, Manipal, Manipal Academy of Higher Education, Manipal, 576104 Karnataka India; 2 Department of Immunohematology and Blood Transfusion, Kasturba Medical College, Manipal, Manipal Academy of Higher Education, Manipal, 576104 Karnataka India; 3 Department of Public Health Dentistry, Amrita School of Dentistry, Amrita Vishwa Vidhyapeetham, Kochi, Kerala India; 4 Department of Prosthodontics, JSS Dental College & Hospital, JSS Academy of Higher Education & Research, Mysuru, Karnataka India

**Keywords:** Reliability generalization, Internal consistency, Oral health, Quality of life, Questionnaire

## Abstract

**Objective:**

To evaluate the pooled estimates of Cronbach’s alpha of the Oral Impacts on Daily Performance (OIDP) questionnaire and explore the moderators that could have influenced the overall estimate.

**Materials and methods:**

A systematic search of common databases such as PubMed, Scopus, EMBASE, and CINAHL was performed from inception till 13th December 2024. Studies in English and those that reported Cronbach’s alpha values for the OIDP questionnaire were included. Studies reported as letters, conference proceedings, or abstracts; secondary analysis of the previous data; studies with alpha values reported for pilot studies; modified versions of the OIDP questionnaires; induced reliability estimates; retracted articles; short communications; and commentaries were excluded. Two review authors independently screened the publications. The information collected included year of publication, country, sample size, age, sex distribution, target population, language of administration, mode of administration, study setting, study design, patient selection, Cronbach’s alpha, and the number of items in the questionnaire. The risk of bias assessment was performed via the COSMIN checklist. Reliability Generalization Meta-analysis was performed via the random effects model (restricted maximum likelihood method) to obtain a pooled untransformed Cronbach’s alpha.

**Results:**

A total of 1069 publications were available for screening, and 54 publications yielded 63 estimates with a sample size of 92,564 (sample size range: 47–12647). The overall pooled Cronbach’s alpha was 0.82 (95% CI = 0.8–0.84), with high heterogeneity (I^2^ = 99.75%; Q = 26702.91). Meta-regression revealed no effects of moderators such as sex (coefficient: 0.02), age (coefficient: 0), language (coefficient: 0), population type (coefficient: 0), continent (coefficient: -0.02), or mode of administration (coefficient: -0.03) on the overall estimate.

**Conclusion:**

The overall estimate of the Cronbach alpha for OIDP questionnaire was above the accepted benchmark. There was no effect of moderators such as sex, age, language, population type, continent, or mode of administration on the overall estimate.

**Supplementary Information:**

The online version contains supplementary material available at 10.1186/s12903-025-05496-3.

## Introduction

Conventional oral health assessment includes clinical oral health assessment by healthcare providers. Considering the impact of oral conditions on daily activities, the concept of oral health-related quality of life (OHRQoL) has gained importance and acceptance among individuals, healthcare providers, and stakeholders. It is a multidimensional concept that incorporates survival; illness and impairment; social, psychological, and physical function; disability; oral health perceptions; opportunity; and interactions between the domains [1].

Various questionnaires have been proposed to quantify OHRQoL in the literature, including the Geriatric Oral Health Assessment, Oral Health Impact Profile, Dental Impact on Daily Living, and Oral Impacts on Daily Performances (OIDP) [2]. OIDP has been extensively researched to establish validity, reliability, and cross-cultural and linguistic validity [2–11]. 

The OIDP questionnaire was developed by Adulyanon in Thailand. It is used to assess an individual’s perception of oral impacts [12]. It measures oral impacts that are based on physical performance (eating, cleaning, speaking, and performing physical activities), psychological performance (sleeping and relaxing, smiling and emotional stability), and social performance (social contact) over the last six months. The responses were on a 5-point Likert scale ranging from strongly disagree to strongly agree. The OIDP questionnaire has been validated among diverse populations and age groups. It has been used in a variety of oral conditions, such as traumatic dental injuries [13, 14], caries [15, 16], gingivitis [17], periodontitis [18–20], malocclusion [17], toothache [20–22], oral mucosal lesions [23, 24] dental anxiety [25], temporomandibular disorders [26] prosthetic treatment need [5, 27], cleft lip and palate [28] self-perceived oral conditions [16, 29–33] and different settings. It has been translated into many languages and has good cross-cultural validity and internal consistency [2–11].

Systematic reviews, reliability generalization (RG), and quality assessment reviews of various OHRQoL instruments have been reported in the literature [34–39]. However, there was no systematic evaluation of the pooled estimates of the internal consistency reliability of the OIDP questionnaire. Reliability is an integral part of the questionnaires. It helps us to contextualize the practical impact of the results on the choice of questionnaires or instruments for research and clinical practice. There are a variety of procedures which are available to estimate the internal consistency reliability of questionnaires, of which Cronbach’s alpha is a popular metric. Cronbach’s alpha can vary with population, language, number of items, and disease conditions and hence there is a need to estimate the overall consistency of a questionnaire.

Reliability generalization meta-analysis (RGMA) was developed to pool the reliability estimates of the questionnaires obtained from various studies. Hence, we aimed to pool the estimates of Cronbach’s alpha of the OIDP questionnaire and explore the moderators that could have influenced the overall estimates via reliability generalization meta-analysis.

## Materials and methods

This systematic review and meta-analysis was reported as per the guidelines of the Reporting Quality of Reliability Generalization Meta-Analyses (REGEMA). The protocol was registered in INPLASY (INPLASY202410060) [40].

### Search strategy

A systematic search of common databases such as PubMed, Scopus, EMBASE, and CINAHL was performed from inception till 13th December 2024. A combination of search terms and free text was used (“oral impacts on daily performance OR OIDP”) on the basis of the previous RGMA of the Child-OIDP [37].

### Inclusion and exclusion criteria

Studies in English and those that reported Cronbach’s alpha values for the OIDP questionnaire were included. Studies reported as letters, conference proceedings, or abstracts; secondary analysis of the previous data; studies with alpha values reported for pilot studies; modified versions of the OIDP questionnaires; induced reliability estimates; retracted articles; short communications; and commentaries were excluded. Observational studies, unlike clinical trials often do not have registries and access to protocol. Similarly, subscriptions to non-English publications are usually limited and inaccessible and require substantial resources for translation. Hence, unpublished and non-English studies were excluded.

### Screening and data extraction

The search results obtained through various databases were added to Rayyan, a web-based tool (https://rayyan.qcri.org/). The title and abstracts were independently screened by two review authors (KCP and VK; Kappa statistic = 0.92). Two review authors independently screened the full texts of the eligible studies (Kappa statistic = 0.93). Discrepancies, if any, were resolved by the third review author (CD). The following data were extracted from the included studies: authors, year of publication, country, sample size, age, sex distribution, target population, language of administration, mode of administration, study setting, study design, patient selection, Cronbach’s alpha, and the number of items in the questionnaire. The acceptable benchmark for Cronbach’s alpha is > 0.7 [41]. The above moderators or study level variables were chosen as they were the readily available from published studies as per the STROBE guidelines. These variables may systematically exhibit a relevant association to the overall estimate. For example, language and geographic location can affect the definition and perception of conceptual words. Comprehension and disease prevalence may also vary with increasing age. Owing to the above reasons, these factors were used to explore their role in heterogeneity.

All the eligible articles were subjected to risk of bias assessment via the COSMIN checklist for internal consistency (Box 4) [42]. Studies were evaluated via 3-items on design requirements (“Was an internal consistency statistic calculated for each unidimensional scale or subscale separately?”), statistical methods (“For continuous scores: Was Cronbach’s alpha or omega calculated?”) and Others (“Were there any other important flaws in the design or statistical methods of the study?”). These were rated on a four-point scale (“very good”, “adequate”, “doubtful” or “inadequate”). An overall score for each study was assigned by taking the lowest score for any of the items (worst score count method).

### Statistical analysis

RGMA was performed via Jamovi software (Version 1.2 https://www.jamovi.org) [32]. I^2^ and Q statistics were used to assess the heterogeneity among the included studies. MA was performed via the random effects model (restricted maximum likelihood method) to obtain a pooled untransformed Cronbach’s alpha. Subgroup analysis was performed on the basis of geographic location, study setting, language, and risk of bias. Publication bias was assessed via Egger’s regression test, and the funnel plot was plotted with the coefficient of alpha on the x-axis and the inverse standard error on the y-axis. Moderator analysis was performed using Meta-regression (mixed effects model) which predicts the study’s effect size with study level variables and uses both fixed and random effects.

## Results

**Fig. 1:**
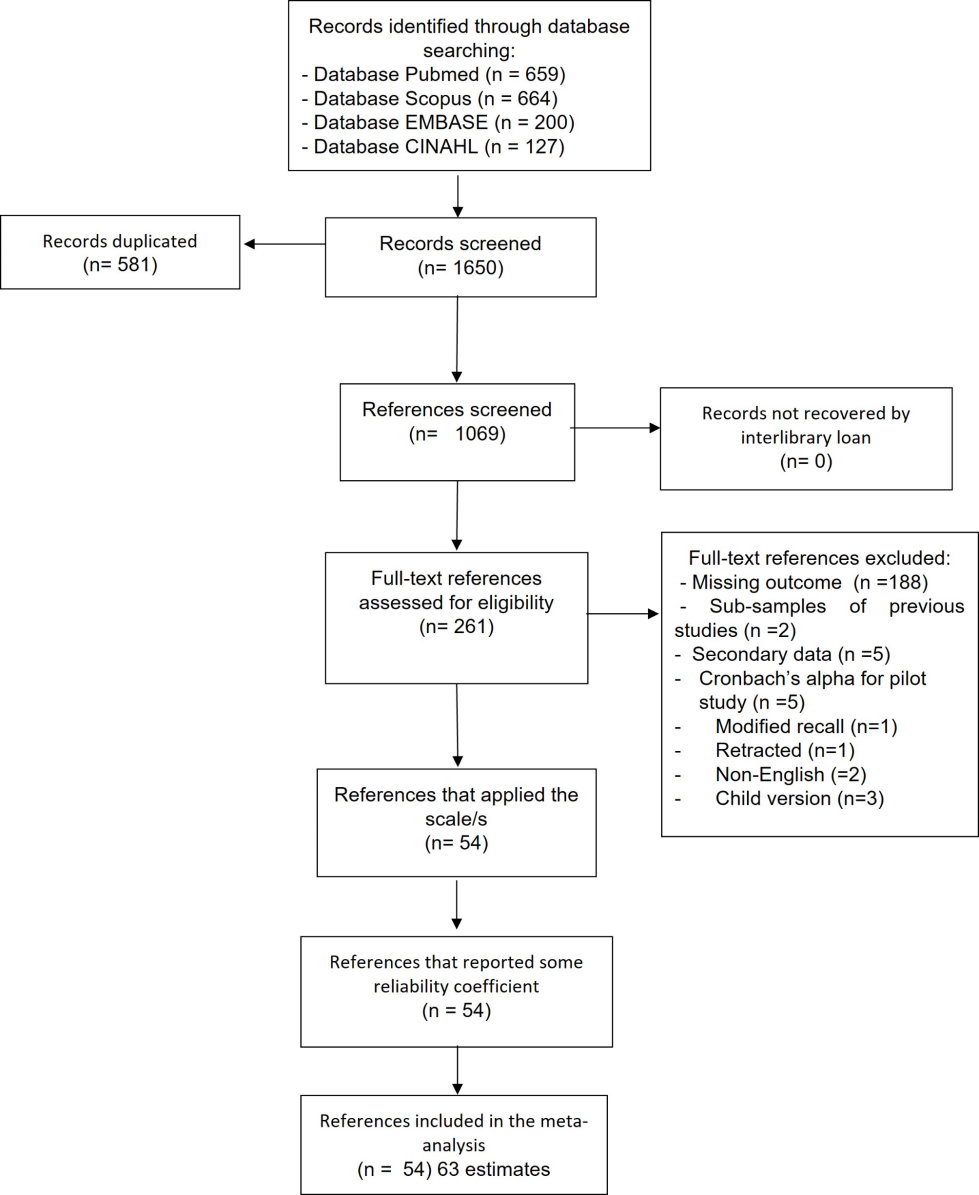
REGEMA flowchart

The search yielded a total of 1650 publications from various databases. After the removal of duplicates (*n* = 581), 1069 publications were available for title and abstracts screening. Only 261 publications were eligible for full-text screening, of which 207 were excluded for various reasons. Data extraction was performed for 54 publications, which yielded 63 estimates (Fig. 1).

The Cronbach’s alpha for most of the studies was above the benchmark (> 0.7). Only three studies reported a Cronbach’s alpha of 0.69 [21, 43, 44].

### Age distribution

The age of the participants ranged from 10 to 105 years. Thirteen estimates did not report the age ranges [11, 16, 25, 30, 45–52], and 29 estimates did not report the mean or median age of the participants [3, 6, 8, 10, 13, 15, 19–21, 26, 27, 33, 44, 52–59]. (Table 1).

**Table 1:** Characteristics of the included studies

Author, Year	Continent	*N*	Mean age	Male	Language	Sampling	number of items	α
Tsakos et al. (1) 2001	Eu	735		382	English	R	10	0.69
Tsakos et al. (2) 2001	Eu	681		232	Greek	R	10	0.77
De Souza et al. 2002	SA	204		123	Portugese	R	8	0.7
Robinson et al. 2003	Eu	165	36		English	C	8	0.88
Masalu et al. (1) 2003	AF	1123		761	English	C	8	0.83
Masalu et al. (2) 2003	AF	228		128	English	C	8	0.87
Astrom et al. 2003	AF	1146			English	R	8	0.9
Soe et al. 2004	Asia	543		254	Burmese	R	8	0.72
Astrom et al. 2005	Eu	1309	43.4	661	Norwegian	R	8	0.8
De Oliveira et al. 2006	SA	504	24		Brazilian	C	9	0.87
Kida et al. (1) 2006	AF	508		218	Kiswahili	R	8	0.83
Kida et al. (2) 2006	AF	512		260	Kiswahili	R	8	0.9
Naito et al. 2007	Asia	47	69	19	Japanese	C	10	0.77
Dorri et al. 2007	Asia	285	36.9	162	Persian	C	11	0.79
Montero et al. (1) 2008	Eu	253	55.9	100	Spanish	R	8	0.78
Montero et al. (2) 2008	Eu	561	43.2	270	Spanish	R	8	0.78
Jung et al. 2008	Asia	668	75.5	328	Korean	R	10	0.85
Hobdell et al. (1) 2009	NA	154		61	Spanish	C	9	0.71
Hobdell et al. (2) 2009	AF	194		86	Afrikaans	C	9	0.86
Hobdell et al.(3) 2009	Eu	177		61	English	C	9	0.72
Wandera et al. 2009	AF	877	25.6		Lumaasaba	C	7	0.81
Astrøm et al. (1) 2010	Eu	6078		2996		R	8	0.9
Astrøm et al. (2) 2010	Eu	4211		2122		R	8	0.89
Thelen et al. 2011	Eu	493	17.2	316	Albanian	R	8	0.77
Costa et al. 2011	SA	116		32		C	8	0.83
Montero et al. 2011	Eu	270	45.2	123	Spanish	C	8	0.74
Harsh et al. 2012	Asia	70	20.91	27	English	C	8	0.79
Purohit et al. 2012	Asia	312	39	112	Kannada	C	8	0.7
Suliman NM et al. 2012	AF	544	37.1	272	Arabic	C	8	0.89
Masalu et al. 2012	AF	1759		863	Kiswahili	R	10	0.99
Erić et al. 2012	Eu	231		116	Serbian	C	10	0.82
Lawal et al. 2013	AF	204	40.9	101	Yoruba	C	8	0.811
Nasir et al. 2013	AF	1262	30.7	548	Arabic	C	7	0.82
Hongxing et al. 2014	Asia	5608	17.2	2692	Chinese	R	8	0.75
Peker et al. 2014	Eu	1324	37.3	512	Turkish	C	8	0.737
Gülcan et al.(1) 2014	Eu	4211		2047	Norwegian	R	8	0.89
Gülcan et al.(2) 2014	Eu	6078		2998	Swedish	R	8	0.89
Hvaring et al. 2014	Eu	163	12.90	80	Norwegian	C	8	0.79
Lawal et al. 2015	AF	234	41.5	131		C	8	0.821
Abegg et al. 2015	SA	200	60.2	117	Brazilian	C	12	0.69
Nair et al. 2016	Asia	202	75	59	Chinese	C	7	0.75
Yeh et al. 2016	Asia	135		107	Taiwanese	C	9	0.94
Lajnert et al. 2016	Eu	702	41.2	255	Croatian	C	8	0.8
Cavalheiro et al. 2016	SA	720		304	Portugese	R	11	0.69
Vettore et al. 2016	SA	4594		1373	Brazilian	R	8	0.816
Chalub et al. 2017	SA	9564		3500		R	9	0.816
Alwadi et al. 2017	SA	3854		1792	Brazilian	R	9	0.78
Chou et al. 2017	Asia	60	42.88	26	Taiwanese	C	9	0.89
Corrêa et al. 2018	SA	96	29.4	36	Brazilian	C	8	0.818
Nagarajappa et al. 2018	Asia	800		536	Hindi	R	10	0.82
Saxena et al. 2018	Asia	414	40.5	158	Hindi	C	9	0.76
Ferreira et al. 2019	SA	5753		1862	Brazilian	R	9	0.856
Mohamed et al. 2019	SA	5445	16.86	2630	Brazilian	R	9	0.78
Kimmie et al. 2021	AF	1615	52	408	English or Afrikaans	C	10	0.96
Birungi, N et al. 2021	AF	345			Lumasaba	C	8	0.91
Techapiroontong et al. 2022	Asia	110	65	44	Thai	C	8	0.76
do Carmo et al. 2022	SA	342	35	276	Brazilian	C	8	0.87
Lim et al. 2022	Asia	368	28.6	127	Malay	C	8	0.75
Andreassen et al.(1) 2022	Eu	216		126	Norwegian	C	8	0.82
Andreassen et al.(2) 2022	Eu	12,647		6045	Norwegian	C	8	0.93
Åstrøm et al. 2022	Eu	164	45	126		C	8	0.87
Yiemstan et al. 2023	Asia	69	55.1	55	Thai	C	8	0.81
Aardal et al. 2023	Eu	107	36	32	Norwegian	C	8	0.91

N: sample size; α: Cronbach’s alpha; C: Convenience; R: random; Eu: Europe; SA: South America; NA: North America; AF: Africa

### Sex distribution

Only two studies did not report the sex distribution of the population included in the calculation of Cronbach’s alpha [8, 60]. Three studies included only females [15, 47, 61]. A total of 40,188 males and 50,972 females were included in this review (Table 1).

### Study setting and design

All the included studies were cross-sectional except for one study [52], and 37 estimates were conducted in school or community settings [3–6, 8–10, 13, 14, 16, 17, 19–21, 26, 27, 29, 33, 44, 48, 50, 52–55, 58, 59, 62]. The cumulative alpha for the school- or community-based settings was 0.82 (Table 2).

**Table 2:** Sub-group analysis of the pooled estimates of Cronbach’s alpha

	*N*	Estimate	SE	95% CI
Continent				
Europe	21	0.82	0.02	0.79–0.85
Asia	15	0.79	0.02	0.76–0.83
Africa	14	0.87	0.02	0.84–0.9
South America	12	0.8	0.02	0.76–0.83
Language				
Others	50	0.81	0.01	0.8–0.84
English	7	0.81	0.03	0.75–0.88
Study setting				
School or community	37	0.82	0.01	0.79–0.84
Others	26	0.82	0.01	0.8–0.85
Mode of administration				
Interview	39	0.81	0.01	0.79–0.83
Self	22	0.84	0.02	0.81–0.87

### Geographic location

Most of the estimates were reported from Europe (*n* = 21), followed by Asia (*n* = 15), Africa (*n* = 14), and South America (*n* = 12). The cumulative alpha values for studies from Europe, Asia, Africa, and South America were 0.82, 0.79, 0.87, and 0.8, respectively (Table 2).

### Language

Six estimates have not reported the language of administration of the OIDP [11, 26, 45, 55, 59] explicitly. One study used both Afrikaans and English versions of the OIDP [50]. The OIDP questionnaire was translated into different languages, including Greek [44], Portuguese [13, 21], Burmese [3], Norwegian [4, 25, 33, 52, 63], Brazilian [17, 19, 20, 22, 28, 43, 47, 58], Kiswahili [54, 56], Japanese [62], Persian [29], Spanish [27, 48, 49], Korean [5], Afrikaans [27], Lumaasaba [15, 61], Albanian [14], Kannada [31], Arabic [24, 64], Serbian [10], Yoruba [65], Chinese [7, 9], Turkish [32], Swedish [33], Taiwanese [46, 57], Croatian [66], Hindi [6, 18], Thai [23, 51], Malay [16] and English [8, 27, 30, 44, 50, 53, 60]. There was not much variation in the pooled estimates among studies that used English versus other languages. (Table 2).

### Risk of bias

All the studies had a low risk of bias.

### Meta-analysis and meta-regression

**Fig. 2:**
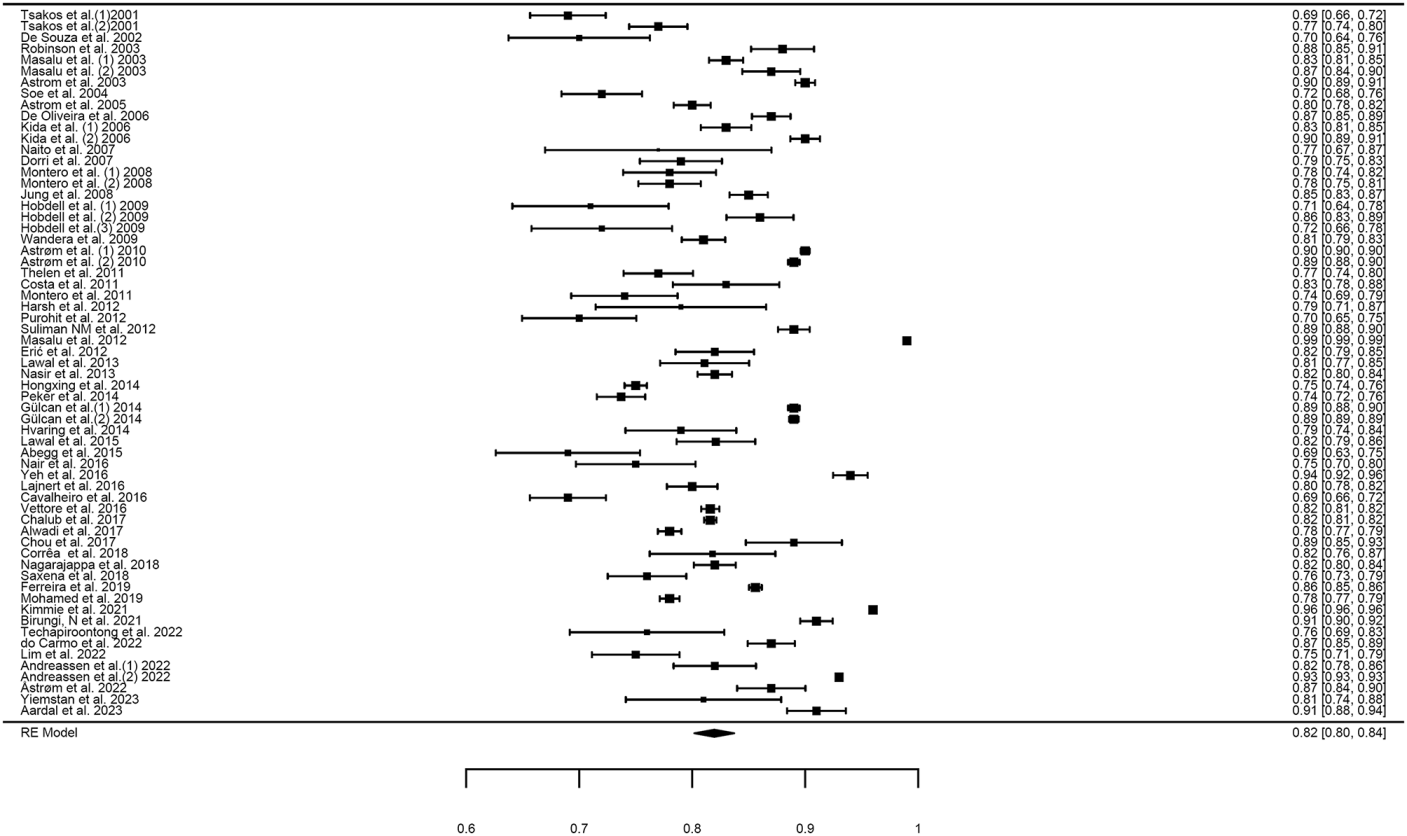
Forest plot showing the cumulative Cronbach’s alpha

A total of 63 estimates were obtained from 54 studies, which yielded a total sample size of 92,564 (sample size range: 47–12647). The random effects model was used with a restricted maximum likelihood method to estimate the pooled Cronbach’s alpha. The overall pooled Cronbach’s alpha was 0.82 (95% CI = 0.8–0.84), with high heterogeneity (I^2^ = 99.75%; Q = 26702.91) (Fig. 2). Meta-regression (mixed-method model) revealed no significant effects of moderators such as sex (coefficient: 0.02; 95% CI: 0.0–0.04), age (coefficient: 0; 95% CI: 0–0), language (coefficient: 0.0; 95% CI: -0.06–0.06), population type (coefficient: 0; 95% CI: -0.04–0.04), continent (coefficient: -0.02; 95% CI: -0.07–0.02) or mode of administration (coefficient: -0.03; 95% CI: 0.07–0.01) on the overall estimate (Table 3).

**Table 3:** Moderator analysis using Meta-regression

	*N*	Coefficient	*p*-value	95%CI	*R* ^2^
Sex†	58	0.02	0.116	0.0-0.04	2.68
Mean age	34	0.00	0.981	0.0–0.0	0
Language	57	0.00	0.975	-0.06–0.06	0
Population type	63	0.00	0.95	-0.04–0.04	0
Continent	63	-0.02	0.309	-0.07–0.022	16.54
Mode of administration	61	-0.03	0.124	0.07 − 0.01	2.28

†: Male: female ratio

### Publication bias

**Fig. 3:**
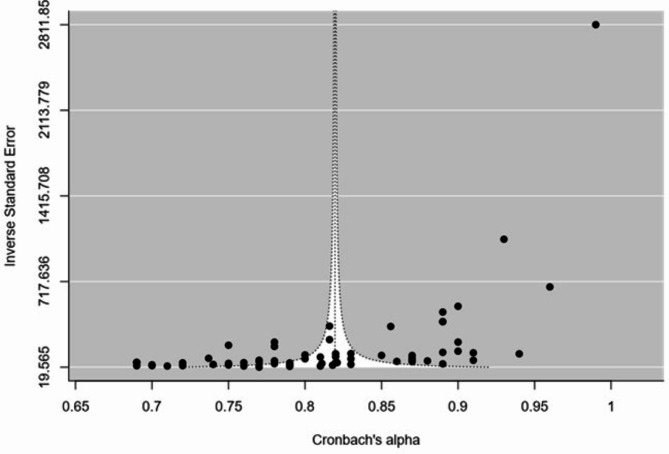
Funnel plot for the assessment of Publication bias

Egger’s regression test (coefficient=-5.76; *P* < 0.001) and the funnel plot showed publication bias. (Fig. 3).

## Discussion

RGMA has many advantages, including consolidating the alpha across studies and populations and estimation of the extent of variation in alpha scores due to different moderators [67]. Since the OIDP has been used in a variety of oral conditions and was shown to be a valid and reliable measure to assess OHRQoL, it was worthwhile to estimate the overall reliability estimates. Cronbach’s alpha is an acceptable metric for estimating internal consistency but can vary with population and oral conditions. Hence, this RGMA aimed to pool the estimates of Cronbach’s alpha obtained from various studies published in the literature.

RGMA has been conducted on the scales that assess OHRQoL [38–40]. However, no attempt has been made to evaluate the reliability estimates of the OIDP questionnaire. In this review, a total of 92,564 individuals from 63 estimates were included. The pooled alpha was 0.82, which suggested that the OIDP questionnaire was a reliable instrument on the basis of the cutoff proposed by Nunnally [41]. Some researchers have recommended a cutoff of 0.8 for research and 0.9 for clinical use [68, 69]. Nevertheless, the pooled estimate was above the recommended values. The pooled estimate of the OIDP questionnaire was higher than that of the child-OIDP questionnaire (0.73) [40], but lower than that of the Child Oral Health Impact Profile questionnaire (0.87) [39]. However, high heterogeneity, similar to our findings, was also reported. Asian studies presented lower pooled alpha values, whereas African studies presented higher pooled alpha values. An attempt was made to evaluate the sources of heterogeneity through subgroup analysis and meta-regression. None of the factors evaluated caused heterogeneity, and a large sample size could have caused heterogeneity. Similar results were reported concerning the child-OIDP questionnaire. Due to this heterogeneity the overall estimate may have been over or under-estimated. Few studies have reported Cronbach’s alpha for subsamples separately due to the difference in the population characteristics. Hence data related to these subsamples were extracted separately to explore the possible heterogeneity and the effect of other moderator variables.

Systematic reviews on observational studies generally have numerous studies conducted among diverse populations and with different characteristics resulting in high heterogeneity. This implies a possible variation in effect size across populations due to potential moderators that may have the effect on the overall estimate. Sub-group analysis or meta-regression helps us to understand the role of these moderator variables. Such variables if significant can have potential to influence in variety of clinical settings.

Many studies were excluded because of the lack of reporting of alpha or alpha values being reported for pilot studies or the use of Cronbach’s alpha values from other studies. It is recommended that alpha values be reported as variables such as population type, scaling of the questionnaire, and distribution of the conditions studied could have influenced the estimates of the alpha. The estimates from this study reinforce the psychometric properties of the OIDP questionnaire.

The inclusion of only published studies and those published in the English language due to limited resources are some of the limitations of this review. Additionally, only a few moderators were evaluated on the basis of the preliminary evaluation of the included publications. Many studies have not reported the mean age of the participants, due to which the role of age as a moderator could not be completely assessed. Studies which are of low quality are less likely to be published. Also, studies with low reliability estimates (below 0.7) may fail to report or less likely to be published and a validated questionnaire may not have studies below the benchmark leading to unavoidable publication bias. Due to these reasons, the overall estimates may vary systematically and there is a possibility of over or underestimation.

### Implications for future research

Future studies should report the reliability estimates of their sample rather than induce reliability through previous research. Other forms of reliability estimates need to be addressed in primary studies to assess the temporal stability of this questionnaire. Standard reporting guidelines need to be followed while the estimates are reported. OIDP has a potential to be incorporated as a patient reported outcome. As the overall estimate is well above the standard benchmark, it can be applied in diverse clinical conditions among individuals of different cultural backgrounds.

## Conclusion

The overall estimate of the Cronbach alpha for OIDP questionnaire was above the accepted benchmark. There was no effect of moderators such as sex, age, language, population type, continent, or mode of administration on the overall estimate.

## Electronic supplementary material

Below is the link to the electronic supplementary material.

Supplementary Material 1

Supplementary Material 2

## Data Availability

All supporting data for this review are included within the manuscript.

## Abbreviations

CI: Confidence interval
COSMIN: Consensus-based standards for the selection of health measurement instruments
OIDP: Oral impacts on daily performance
OHRQoL: Oral health related quality of life
RGMA: Reliability generalization meta-analysis
REGEMA: Reporting quality of reliability generalization meta-analyses
