# Supplementary material for: Reliability generalization meta-analysis of Cronbach’s alpha of the oral impacts on daily performance (OIDP) questionnaire
Source: BMC Oral Health. 2025 Feb 11;25:220. doi: 10.1186/s12903-025-05496-3 (PMC11817562; doi:10.1186/s12903-025-05496-3)
Supplement: Supplementary file 1 — Supplementary Material 1 [file 12903_2025_5496_MOESM1_ESM.docx]

**REGEMA Checklist**

| **TITLE** |  | **Yes** | **No** | **Unclear** | **NA** |
| --- | --- | --- | --- | --- | --- |
| *1. Title* | In the title include: (a) the term “reliability generalization” or “meta-analysis” together with some explicit indication to reliability (internal consistency, test-retest, inter- or intra-rater) and (b) the name of the scale or, if more than one scale, the attribute/outcome measure that the scales are assessing. | **✓** |  |  |  |
| **ABSTRACT** |  | **Yes** | **No** | **Unclear** | **NA** |
| *2. Abstract* | In the abstract explicitly state: (a) that the objective was to carry out a reliability generalization (RG) meta-analysis of one or several scales; (b) eligibility criteria of the studies; (c) data sources with the temporal range covered; (d) types of reliability coefficients analyzed; (e) statistical model applied; (f) main results (e.g., pooled reliability coefficient and 95% CI, moderator variables related to reliability); and (g) main conclusions. In case of space limitation, (b) and (c) criteria can be omitted. | **✓** |  |  |  |
| **INTRODUCTION** |  | **Yes** | **No** | **Unclear** | **NA** |
| *3.* *Background* | In the background include: (a) a conceptual definition of the attribute/outcome measure assessed by the scale/s; (b) description of the target population/s to which the scale/s is/are applied and its/their purposes (e.g., screening, clinical diagnosis); (c) a complete description of the scale/s (length, number of categories), including the versions and adaptations to other languages/cultures; and (d) a brief presentation of reliability estimates obtained in previous psychometric studies of the scale/s. Optionally, a brief review of validation studies of the scale/s (e.g., exploratory/confirmatory factor analyses, concurrent/convergent/discriminant validity, responsiveness) could be included. | **✓** |  |  |  |
| *4. Objectives* | State whether the purpose of the meta-analysis was to obtain a more precise overall reliability coefficient estimate and/or investigate how reliability coefficients vary among different applications of the scales. Optionally, specify whether one objective of the meta-analysis is to estimate the reliability induction rates of the scale/s. | **✓** |  |  |  |
| **METHOD** |  | **Yes** | **No** | **Unclear** | **NA** |
| 5. *Selection criteria* | Specify inclusion criteria: (a) name/s of the scale/s analysed in the RG meta-analysis, as well as the versions and/or adaptations included; (b) geographical and/or cultural restrictions; (c) years considered; (d) language of the paper; (e) publication status; (f) to report any reliability estimate based on the study-specific sample/s; (g) type/s of reliability considered (e.g., internal consistency, temporal stability, inter-/intra rater reliability…); (h) target population/s (e.g., community, clinical, subclinical/analog, university…); and (i) minimum sample size required. | **✓** |  |  |  |
| 6. *Search strategies* | Specify how the studies were located: (a) electronic databases consulted; (b) other formal search procedures (e.g., manual search in specific journals, backward search from references listed in selected studies); and (c) informal search procedures (e.g., internet searches, contacting study authors to identify additional studies). For electronic searches, describe the search strategy, including the keywords used and how they were combined, and the search limits (e.g., fields where the keywords were searched - title, abstract, full-text -, temporal range, language). | **✓** |  |  |  |
| 7. *Data extraction* | Describe the characteristics extracted from the studies, including: (a) sample size/s, mean/s and standard deviation/s of total test scores and subscales (if applicable); (b) sample characteristics (e.g., target population, country, mean age, standard deviation of the age, gender distribution, ethnic distribution, disorder history −mean and SD in years); (c) test version (e.g., adaptation/version, number of items, reporting format −self-report, clinician); (d) methods (e.g., study design, purpose of the study −psychometric versus applied−, quality checklist); (e) extrinsic characteristics (e.g., publication status, researchers’ affiliations, funding source). | **✓** |  |  |  |
| *8. Reported reliability* | Identify the types of reliability coefficients included in the RG meta-analysis: internal consistency (e.g., Cronbach’s alpha, KR-21, parallel forms, omega), temporal stability (test-retest), inter- and intra-rater reliability (e.g., intraclass correlation, kappa coefficient). Clearly state that separate meta-analyses were conducted for each type of reliability coefficient. In case of applying a multivariate/MASEM approach, specify the type of statistical information extracted from the studies (i.e., item-item correlation/covariance matrices, factor loadings, etc.). | **✓** |  |  |  |
| *9. Estimating the reliability induction and other sources of bias* | In case that the meta-analysis intends to estimate the reliability induction, identify the types of reliability induction: induction by omission (no mention of test reliability whatsoever) or reporting induction (vague or precise reporting). Describe how other sources of bias were assessed (e.g. assumptions of the reliability coefficient, adequacy of the measurement model, etc.). |  |  | **✓** |  |
| *10. Data extraction of inducing studies* | Declare whether characteristics of inducing studies were also extracted or if, on the contrary, only characteristics of studies that reported reliability were extracted. | **✓** |  |  |  |
| *11. Reliability of data extraction* | Describe how the reliability of data extraction process was appraised: how many coders which agreement coefficients were applied (e.g., kappa coefficient, intraclass correlation), which values were obtained, and how disagreements were dealt with. | **✓** |  |  |  |
| *12. Transformation method* | State whether or not the reliability coefficients were transformed for the meta-analytic integration. If relevant, specify the transformation methods: Fisher´s Z for correlation coefficients (e.g., test-retest coefficients), Bonett’s and Hakstian and Whallen’s transformation for internal consistency coefficients (e.g., Cronbach’s alpha), reliability index, measurement error (e.g., standard error of measurement), or other (specify). | **✓** |  |  |  |
| *13. Statistical model* | Describe the statistical model(s) assumed in the meta-analytic integration for estimating the average reliability coefficient and for analysing the influence of moderator variables (e.g. fixed-effect(s), random-effects, mixed-effects, varying-coefficient models, generalized linear models), as well as the analysis framework (frequentist or Bayesian). In case of applying a multivariate/MASEM approach, describe how the item correlation/covariance matrices or factor loadings were synthesized. | **✓** |  |  |  |
| *14. Weighting method* | Specify the weighting method applied in the meta-analytic integration: unweighted, weighting by sample size, weighting by inverse variance, or other weighting methods. | **✓** |  |  |  |
| *15. Heterogeneity assessment* | Describe how heterogeneity among reliability coefficients was assessed (e.g., standard deviation, *Q* statistic, *I*^2^ index, between-studies variance, 75% rule of Hunter-Schmidt). If relevant, specify the between-studies variance estimator (DerSimonian and Laird, Maximum Likelihood, Restricted Maximum Likelihood, Empirical Bayes, Paule and Mandel), as well as how confidence intervals, credibility intervals, or prediction intervals were calculated. | **✓** |  |  |  |
| *16.* *Moderator analyses* | If relevant, describe how the influence of moderator variables was assessed (e.g., subgroup analyses, meta-regression analyses, correlational analyses). | **✓** |  |  |  |
| *17. Additional analyses* | Describe other additional analyses accomplished, such as sensitivity analyses (e.g., statistical analyses with transformed and untransformed reliability coefficients, one-to-one deleting of reliability coefficients, assessment of publication bias, reporting biases, and other sources of bias). | **✓** |  |  |  |
| *18. Software* | Mention the software and version used to carry out the statistical analyses (e.g., metafor in R, Proc MIXED in SAS, Comprehensive Meta-analysis). | **✓** |  |  |  |
| **RESULTS** |  | **Yes** | **No** | **Unclear** | **NA** |
| *19. Results of the study selection process* | Describe, ideally with a flow chart, the selection process of the studies, specifying the number of studies identified from each search source, excluded studies and reasons why, and the number of studies that reported and induced reliability of test scores. Regarding reliability induction, report induction rates, distinguishing between induction “by omission” and “by report” (see e.g., REGEMA flowchart). Furthermore, it is advisable to compare the reliability induction rates as a function of variables such as publication year, country/continent and study purpose (psychometric vs. applied). | **✓** |  |  |  |
| *20. Mean reliability and heterogeneity* | Present pooled reliability coefficients and confidence/credibility intervals for the scale (and subscales, if applicable) and for each type of reliability (e.g., internal consistency, temporal stability, inter- and intra-rater agreement). In case of applying any transformation of the reliability coefficients, results should be back-transformed to the original metric to facilitate interpretation. Illustrate the distribution of reliability coefficients with graphical techniques (e.g., forest plots, box plots, stem and leaf displays, histograms) and describe the degree of heterogeneity by one or more heterogeneity measures (see Item 15). | **✓** |  |  |  |
| *21. Moderator analyses* | For categorical moderators, provide the pooled reliability coefficient, confidence interval and other heterogeneity measures for each category of the moderator. For continuous moderators, include the regression coefficients, standard errors and confidence limits. For both types of moderators, report results of the statistical significance tests, misspecification tests, and proportion of variance accounted for. As a further step, it is advisable to fit a predictive/explanatory model including the most relevant moderator variables. | **✓** |  |  |  |
| *22. Sensitivity analyses* | Report or describe the results of any sensitivity analyses conducted (see Item 17). |  |  |  | **NA** |
| *23. Comparison of inducing and reporting studies* | If performed, present the results of comparing the characteristics of inducing and reporting studies (e.g., sociodemographic and clinical characteristics of the samples). |  |  |  | **NA** |
| *24. Data set* | Tabulate the characteristics of the individual studies that reported reliability (see Item 7). Tables can be presented as appendices or supplementary files. In addition, list of all studies included in the RG meta-analysis, either in the reference section or as a supplementary file. | **✓** |  |  |  |
| **DISCUSSION** |  | **Yes** | **No** | **Unclear** | **NA** |
| *25. Summary of results* | Present the main results, such as mean reliability exhibited by the scale/test and moderators of the reliability coefficients. If available, discuss the results in the light of previous evidence. | **✓** |  |  |  |
| *26. Limitations* | Discuss the limitations of the meta-analysis. Include an explicit statement of the reliability induction rates and the extent to which inducing and reporting studies are comparable in terms of samples characteristics. | **✓** |  |  |  |
| *27. Implications for practice* | Provide guidelines for professional practice regarding the usefulness of the scale/test in different settings and target populations. | **✓** |  |  |  |
| *28. Implications for future research* | Include recommendations for researchers regarding the conditions under which the scale/test should be applied. | **✓** |  |  |  |
| **FUNDING** |  | **Yes** | **No** | **Unclear** | **NA** |
| *29. Funding* | State the financial sources of the meta-analysis, as well as potential conflict of interests of the authors. | **✓** |  |  |  |
| **PROTOCOL** |  | **Yes** | **No** | **Unclear** | **NA** |
| *30. Protocol* | State whether a protocol of the meta-analysis was previously published or made accessible in some web-site (e.g., in Prospero). | **✓** |  |  |  |

*Note*. NA: Not Applicable.
