# Supplementary material for: Reliability generalization meta-analysis of Cronbach’s alpha of the oral impacts on daily performance (OIDP) questionnaire
Source: BMC Oral Health. 2025 Feb 11;25:220. doi: 10.1186/s12903-025-05496-3 (PMC11817562; doi:10.1186/s12903-025-05496-3)
Supplement: Supplementary file 2 — Supplementary Material 2 [file 12903_2025_5496_MOESM2_ESM.docx]

| Pubmed | Oral Impacts on Daily Performance OR OIDP AND ((english[Filter]) AND (alladult[Filter])) |
| --- | --- |
| Scopus | TITLE-ABS-KEY ( oral AND impact AND on AND daily AND performances ) OR TITLE-ABS-KEY ( oidp ) AND ( EXCLUDE ( DOCTYPE , "re" ) ) AND ( LIMIT-TO ( LANGUAGE , "English" ) ) AND ( LIMIT-TO ( EXACTKEYWORD , "Humans" ) OR LIMIT-TO ( EXACTKEYWORD , "Human" ) OR LIMIT-TO ( EXACTKEYWORD , "Adult" ) ) |
| EMBASE | ('oral impacts on daily performance'/exp OR oidp) AND [english]/lim AND ([adult]/lim OR [aged]/lim) |
| CINAHL | TX Oral Impact on daily performances OR TX OIDP  Narrow by SubjectAge: - all adult Narrow by Language: - english |
